# Effect of Transepithelial Photorefractive Keratectomy without Mitomycin C in the Treatment of Femtosecond Laser In Situ Keratomileusis Corneal Flap Complications

**DOI:** 10.1155/2021/8847922

**Published:** 2021-01-13

**Authors:** Jing Wang, Weiqian Cao, Liming Tao

**Affiliations:** Department of Ophthalmology, The Second Affiliated Hospital of Anhui Medical University, 678 Furong Road, Hefei 230601, China

## Abstract

**Purpose:**

To assess the efficacy and safety of transepithelial photorefractive keratectomy (TPRK) without mitomycin C as treatment for femtosecond laser in situ keratomileusis (FS-LASIK) corneal flap complications.

**Methods:**

Eight patients with corneal flap complications that occurred after FS-LASIK (five with eccentric flaps, two with buttonhole flaps, and one with a thick flap) were included in the study. Patients were treated with TPRK without mitomycin C between two weeks and twelve months after surgery. The postoperative manifest refraction, uncorrected distance visual acuity, and haze formation were assessed during six months of follow-up.

**Results:**

The mean manifest refractive spherical and cylinder refraction was 0.16 ± 0.26 and −0.44 ± 0.33 diopters, respectively, at six months postoperatively. The uncorrected distance visual acuity was above 20/25 in all patients after six months of follow-up. No haze formation was detected.

**Conclusions:**

TPRK without mitomycin C appears to be a safe and effective treatment for FS-LASIK corneal flap complications.

## 1. Introduction

Laser in situ keratomileusis (LASIK) is frequently used for the surgical correction of ametropia. The creation of a corneal flap is the first and most critical step during LASIK surgery [1]. Biomechanical changes and decreased tensile strength after the surgery may hinder the healing of the corneal flap, resulting in severely reduced adhesion between the flap and the stromal bed, often only between a quarter and half of the normal situation [2]. This loss of adhesion may lead to corneal flap-related complications which occur in 0.20–0.56% of cases [3, 4]. The popularization of the femtosecond laser has reduced the probability of these complications; however, they may occur if the operator is relatively inexperienced. Dislocation of the corneal flap may lead to corneal opacity, irregular astigmatism, ingrowth of the corneal epithelium, and an increase in higher-order aberrations, resulting in a decrease of best-corrected distance vision acuity [5]. Previously, most patients were not treated with immediate surgery but received either corneal lamellar flap reconstruction or corneal surface surgery with mitomycin C (MMC) three months later [6].

The second operation uses a corneal lamellar knife to create the flap. The dissected corneal flap should be larger and deeper, but this does not apply to patients with a thin cornea or high myopia. Transepithelial photorefractive keratectomy (TPRK) is an effective method for the treatment of corneal flap-related complications after LASIK. It does not require the production of a corneal flap, reduces the risk of both wrinkles in the flap and corneal epithelial implantation, and can also deal with the corneal scars produced by the unsuccessful creation of the corneal flap in the first operation. Therefore, it is particularly suitable for patients with relatively thin corneas or for subsequent operations. In addition, it allows the interval between the first and second operations to be relatively short, generally within two to four weeks after the first operation. Furthermore, the incidence of subepithelial fibrosis and haze formation is greatly reduced and the procedure can also deal with irregularities on the corneal surface.

TPRK is often combined with MMC for the treatment of femtosecond postoperative corneal flap complications, using a conventional MMC concentration of 0.2 g/L. Related study has shown that the addition of MMC effectively prevents haze formation [7]. Although there are no reports of serious complications in using MMC in surface ablation surgery, some studies have suggested that it may cause delayed healing of the corneal epithelium, decreased endothelium, and decreased function [8, 9]. In addition, MMC is difficult to obtain in China, so it is not used for the treatment of corneal flap complications. The purpose of this study was to investigate the effect of TPRK surgery without MMC on the treatment of corneal flap complications.

## 2. Patients and Methods

### 2.1. Collection of Data

We retrospectively reviewed data of eight patients (eight eyes) with failed femtosecond laser-assisted valvular surgery for ametropia conducted in the Ophthalmic Treatment Room of the Second Affiliated Hospital of Anhui Medical University from June 2013 to April 2019.

All procedures were conducted according to the tenets of the Declaration of Helsinki and its later amendments. The need to obtain informed patient consent was waived because of the retrospective study design.

All eight patients in the study underwent a comprehensive preoperative ophthalmic examination, including initial corneal topography (Oculus Optikgeräte GmbH, Wetzlar, Germany), manifest and cycloplegic refraction, and slit-lamp microscopic examination of the anterior and posterior segment and retina. Informed consent was obtained from the patients before each procedure.

### 2.2. SCHWIND AMARIS Excimer Laser System

In contrast to the earlier excimer laser photorefractive keratectomy (PRK), TPRK uses the SCHWIND AMARIS excimer laser system (Schwind Eye-Tech Solutions GmbH, Germany). The treatment principle is based on the assumption of a central epithelial thickness of 55 *μ*m and a peripheral epithelial thickness of 65 *μ*m. It is feasible to measure the corneal epithelial thickness before the operation and to input it into the system for personalized settings. The first step is to ablate the refractive degree, while the second step is to ablate the corneal epithelium, making it is similar to phototherapeutic keratectomy (PTK).

### 2.3. Surgical Techniques

In all patients, corneal flaps were created with the Ziemer LDV Z4 femtosecond laser machine (Ziemer Group AG, Port, Switzerland). The corneal flap thickness was 100 *μ*m, and the diameter was 8.5 mm. Among the patients, five had obvious decentered corneal flaps, and in one case, the gasket had been misplaced due to a mistake by the surgical assistant, resulting in a corneal thickness of 250 *μ*m. In two cases, it was found that the stromal layer in the central visual axis had not been incised, and after a second ablation of the thickness and size of the original corneal flap, the flap could not be lifted successfully and the operation was aborted. All the patients' eyes were covered with bandaged contact lenses (PureVision, Bausch & Lomb, Quebec, Canada) after corneal flap re-positioning.

After the operation, we used 0.5% levofloxacin drops, 0.1% flumilone drops, and 0.3% sodium hyaluronate drops (Santen Pharmaceutical, Osaka, Japan) four times daily, removing the bandaged contact lenses three days later. The patients who had received secondary femtosecond corneal flap ablation had corneal epithelial ingrowth and corneal flap dissolution, and the subflap epithelium was curetted one month after the operation. The corneal epithelium was relieved after the operation, but the corrected visual acuity was not improved.

Patients who had received TPRK had stable diopter measurements and intact corneal epithelia, as assessed between two weeks and 12 months after the operation. The surgical procedure was as follows: after irrigating the conjunctival sac with balanced salt solution, the corneal surface was wiped with a damp aseptic sponge to keep the corneal surface moist and smooth. The cornea was ablated by excimer laser, irrigating and cooling the cornea with the balanced salt solution at 4°C, after which the bandaged contact lenses were positioned and kept in place for three days. After the operation, 0.5% levofloxacin was administered four times daily for one week, together with 0.1% flumilone four times daily, reducing the amount once a month, for four months, and 0.3% sodium hyaluronate four times daily for six months.

All patients were followed up at three days, one week, one month, three months, and six months after the operation. The follow-up included assessment of uncorrected visual acuity, optometry, and assessment of the haze score. Haze was graded following the classification of Fantes et al.: grade 0 indicated corneal transparency with no opacity, grade 0.5 showed very slight opacity with slit-lamp oblique illumination inspection, grade 1 indicated corneal opacity that did not affect the observation of the iris texture, grade 2 indicated slightly unclear iris texture, in grade 3, the iris and lens observations were moderately unclear, and in grade 4, the scar area matrix was completely turbid and the anterior chamber was not visible.

## 3. Results

The patients included five males and three females, aged 22.50 ± 3. 66 (range 18–28) years, with corneal thicknesses of 545.62 ± 4.11 (502–627) *μ*m, K1 of 43.63 ± 1.91 (40.85–46.37), K2 values of 42.39 ± 1.30 (40.73–44.88), and spherical lens diopter values of −2.85 ± 2.36 (+2.00 mm) D. The degrees of the cylindrical lens were −1.05 ± 1.32 D. The detailed basic information of all the patients before the operation is listed in Table 1.

**Figure 1:**
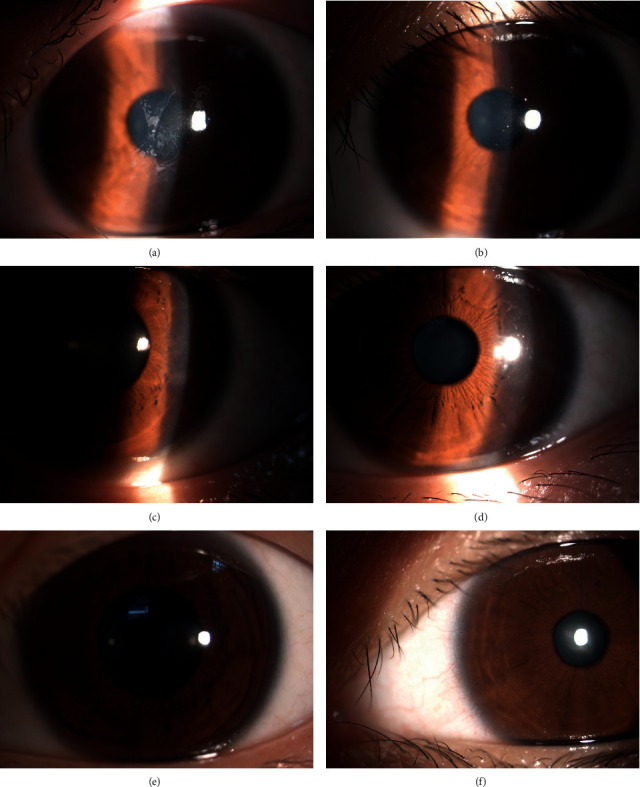
Photographs of the anterior segment of the third patient. (a) shows pre-TPRK, while (b)–(f) show post-TPRK at some follow-up assessments.

**Figure 2:**
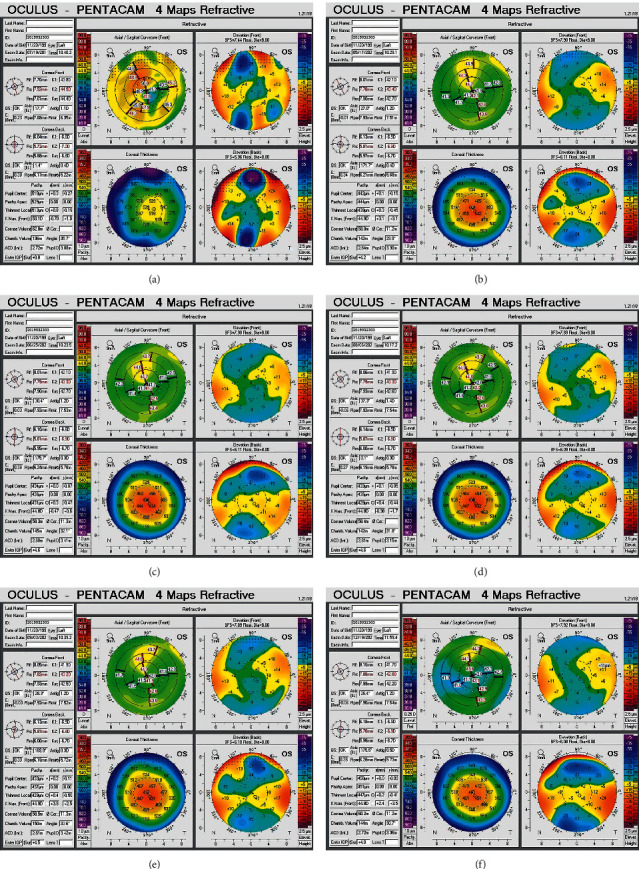
Corneal topography of the third patient. (a) shows pre-TPRK, while (b)–(f) show post-TPRK at some follow-up assessments.

All patients received the TPRK operation between two weeks to 12 months after the original surgery. The diopter, uncorrected visual acuity, and best-corrected visual acuity values before TPRK are shown in Table 2. Fifty percent of the patients had a visual acuity above 20/25 one week after the operation, 87.5% had a visual acuity above 20/25 at one month after the operation, and 100% had a visual acuity above 20/25 at three months after the operation. The postoperative uncorrected visual acuity and spherical and cylindrical lens degrees are shown in Table 2. No haze was found during the six-month follow-up after the operation.  Figure 1 shows the corneal epithelium of the third patient after FS-LASIK and pictures of the anterior segment before and after TPRK.  Figure 2 shows the corneal topography examined by the Pentacam anterior segment analyzer (Oculus Optikgeräte GmbH, Wetzlar, Germany) before and after TPRK, corresponding to Figure 1, which shows the decrease in the corneal scar and restoration of the regular corneal surface after TPRK.

## 4. Discussion

The risk factors for corneal flap complications include celophthalmia, the presence of a steep cornea, conjunctival scarring, small corneal diameter, and conjunctival incarceration during negative pressure suction. In the past, the stromal bed was cleaned carefully to ensure that the corneal flap was properly restored to avoid epithelial ingrowth. The time of reoperation was postponed for at least twelve weeks to ensure that the corneal flap had healed satisfactorily and the diopter was stable. Reoperation methods include laser in situ keratomileusis (LASIK) [10] for deeper corneal flaps, PRK [11], or PTK [12] combined with MMC. Each method of reoperation has risks and potential complications. Deeper corneal flaps may cause intralamellar fragmentation and corneal folds that may lead to a decline in the best-corrected visual acuity, as well as producing glare, halo, and anisometropia which will affect the patients' daily lives and reduce their quality of life and satisfaction with the refractive surgery [13]. Early surface surgical techniques such as PRK, laser subepithelial keratomileusis (LASEK), or epipolis laser in situ keratomileusis (EPI-LASIK) are contraindications. Previous reports have always applied TPRK combined with MMC to solve corneal flap complications [6].

Mitomycin C (MMC) is an antibiotic derived from *Streptomyces caespitosus*. MMC also has antimetabolic effects and can reduce scarring. It forms cross-links with double-stranded DNA leading to an inhibition of replication and DNA breakage. It has been shown to prevent fibroblast proliferation, resulting in the inhibition of scar formation. It is, therefore, widely used in corneal surface refractive surgery to prevent the occurrence of haze. However, Gharaee [14] found that MMC can damage the corneal endothelium, especially in patients with a thin cornea or high myopia who have a thinner corneal stroma after laser ablation. Medeiros [15] also found that when PRK was used with MMC, the toxic effect on corneal nerves was reduced, but this effect was only significant one month after the operation. Hence, in PRK surgery [16], some scholars have found that there is no difference in the uncorrected visual acuity and occurrence and degree of postoperative haze with and without MMC. However, others believe that the use of MMC in the treatment of hyperopia has better predictability and greater curative effect while producing fewer changes in the corneal topography and a lower regression rate of hyperopia [17, 18]. However, when Adib [19] performed surgery in patients with mild to moderate myopia (≤−5.50 diopters (D)), the total ablation depth (including the epithelial and stromal layers) of TPRK was 160 *μ*m or less. Each patient's right eye was treated with 0.02% MMC for 10 seconds while the left eye was not exposed to MMC. It was found that the degree of haze in both eyes was similar, and there was no difference in the postoperative effect. However, the corneal endothelial cell indices of MMC-treated eyes showed a worse profile. Caution should be exercised in the application of MMC in patients with mild to moderate myopia. Thus, it is controversial whether the surface surgery should be combined with MMC. The causes of haze include postoperative roughening of the corneal stroma, delayed epithelial healing, the depth of ablation, correction of high astigmatism, ultraviolet exposure, and genetic effects. On the other hand, TPRK results in a smooth corneal stroma, a shorter epithelial healing time, a lower degree of pain, and less incidence of postoperative haze than traditional surface surgery [20–22]. Most of our patients had low to moderate myopia or hyperopia requiring less cutting depth, so good therapeutic effects were still achieved without the combination with MMC.

It has been reported that TPRK can treat high-order aberrations and irregular astigmatism after keratoplasty [23], can be combined with corneal cross-linking surgery for ametropia [24] and keratoconus [25], and can be combined with corneal stroma ring implantation and corneal cross-linking surgery for keratoconus [26], all of which prove that this operation can achieve a good curative effect for some special cases with fewer complications.

## 5. Conclusions

To sum up, compared with LASIK surgery, TPRK has advantages in the treatment of some corneal flap complications, corneal epithelial implantation, and anterior corneal stromal scarring. It can not only correct ametropia but can also treat irregular astigmatism and corneal scarring without the use of MMC.

## Figures and Tables

**Table 1:** Basic information of patients.

Patient	Gender	Age (y)	Eye	Corneal thickness (mm)	K1	K2	Sphere (D)	Cylinder (D)	UDVA	CDVA
1	M	19	Right	627	40.85	40.73	−3.87	−0.37	20/800	20/20
2	F	23	Right	535	43.16	42.77	−4.25	0	20/200	20/20
3	F	28	Left	523	45.45	44.88	−4	0	20/800	20/20
4	M	19	Left	519	44.69	43.17	−4.25	−1	20/250	20/20
5	M	26	Right	502	42.68	41.80	−2.75	−0.5	20/100	20/20
6	M	18	Right	584	41.54	41.11	−0.75	−0.75	20/40	20/16
7	F	25	Right	551	44.29	42.62	−5	−1.75	20/160	20/25
8	M	22	Left	524	46.37	42.02	2	−4	20/40	20/25

D = diopters; UDVA = uncorrected distance visual acuity; CDVA = corrected distance visual acuity.

**Table 2:** Preoperative and postoperative diopter measurements of patients receiving TPRK.

Patient	1	2	3	4	5	6	7	8
Sphere (D) of failed corneal flap	−1.5	−4.25	−3.62	−2.25	−1	−0.5	−5	0.75
Cylinder (D) of failed corneal flap	−1.5	0	−0.75	−4.75	−0.5	−1.75	−2.25	−4.25
UDVA	20/40	20/200	20/800	20/200	20/200	20/100	20/2000	20/40
CDVA	20/25	20/20	20/50	20/200	20/20	20/25	20/25	20/32
1 wk sphere (D)	0.25	−0.75	−1.37	1.25	0.25	−1.12	0.75	−0.5
1 wk cylinder (D)	−0.25	−0.62	−0.62	−1.25	−0.62	−1.12	−0.5	−0.5
1 wk UDVA	20/25	20/32	20/40	20/160	20/32	20/25	20/25	20/25
1 mo sphere (D)	0.37	0	−1.25	0.62	0.37	0.62	0.25	−0.37
1 mo cylinder (D)	0	−0.37	−0.75	−1.37	−0.37	−0.5	−0.25	−0.62
1 mo UDVA	20/20	20/16	20/25	20/40	20/25	20/16	20/20	20/25
3 mo sphere (D)	0.62	0.25	−1.12	0.5	0.25	0.5	0	−0.25
3 mo cylinder (D)	0	0	−0.62	−1.12	−0.25	−0.37	−0.37	−0.75
3 mo UDVA	20/20	20/16	20/25	20/25	20/20	20/16	20/20	20/25
6 mo sphere (D)	0.25	0.37	0	0.37	0	0.37	0	0.25
6 mo cylinder (D)	−0.25	0	−1.25	−1	−0.37	−0.25	−0.25	−0.62
6 mo UDVA	20/20	20/16	20/20	20/25	20/20	20/16	20/20	20/20

D = diopters; UDVA = uncorrected distance visual acuity; CDVA = corrected distance visual acuity; wk = week; mo = month.

## Data Availability

The data used to support the findings of this study are available from the corresponding author upon reasonable request.
